# Sellar–suprasellar pituitary lymphoma mimicking pituitary adenoma: a case report with literature review

**DOI:** 10.1093/jscr/rjaf447

**Published:** 2025-10-20

**Authors:** Ali Almomen, Moath A Alfaleh, Omar Alanzi, Mohsen M Bashammakh

**Affiliations:** Department of Otolaryngology-Head and Neck Surgery, King Fahad Specialist Hospital, Dammam 32253, Saudi Arabia; Department of Otolaryngology-Head and Neck Surgery, King Fahad Specialist Hospital, Dammam 32253, Saudi Arabia; Department of Otolaryngology-Head and Neck Surgery, King Fahad Specialist Hospital, Dammam 32253, Saudi Arabia; Faculty of Medicine, Imam Abdulrahman Bin Faisal University, King Faisal Bin Abdulaziz Road, Al Rakah District, Dammam 34211, Saudi Arabia

**Keywords:** suprasellar lymphoma, sellar lymphoma, primary central nervous system lymphoma, pituitary lymphoma, non-Hodgkin lymphoma

## Abstract

Sellar–suprasellar lymphoma (SSL) is an extremely rare tumor, constituting fewer than 1%. It is a variant of primary central nervous system lymphoma. SSL is usually seen in people around 60 years of age, exhibiting a slight female predominance. Diffuse large B-cell lymphoma represents the most predominant subtype. We report a 57-year-old female with a 9-month history of worsening severe left-sided headaches involving the left eye, associated with nausea, vomiting, blurred vision, and significant unintentional weight loss. Initial imaging revealed a pituitary tumor, and she was diagnosed with a pituitary macroadenoma. She underwent endoscopic trans-sphenoidal debulking. Histopathological examination confirmed a high-grade diffuse large B-cell lymphoma. She was treated with chemotherapy and radiotherapy which reduced the mass and improved her symptoms. Our case underscores the importance of considering SSL, particularly in patients with unusual sellar masses. Effective treatment needs a multidisciplinary approach, integrating radiotherapy and endocrine support when demanded.

## Introduction

Sellar–suprasellar lymphoma (SSL) is a very rare tumor, constituting fewer than 1% of surgically treated pituitary masses [1]. It is a variant of primary central nervous system lymphoma, which affects the brain, spinal cord, or eyes without systemic spread [2]. SSL is usually seen in people around 60 years of age, exhibiting a slight female predominance, and commonly presents with headache, hypopituitarism, and vision disturbances [3]. Notably, most reported cases of SSL are of B-cell origin, with diffuse large B-cell lymphoma representing the predominant subtype [4]. Given its rarity, diagnosis is often difficult and usually confirmed through histopathological analysis [5].

## Case presentation

A 57-year-old female known to have type 2 diabetes mellitus, hypertension, dyslipidemia, and hyperprolactinemia presented with severe, pulsatile left-sided headaches impacting the left eye, rated 10/10 in severity, and associated with lacrimation and blurred vision in the affected eye, nausea, vomiting, and unintentional weight loss of 20 kg over 9 months. Afterward, her blurry vision progressed to left temporal hemianopia accompanied by intermittent fevers, night sweats, and fatigue. The initial neurological examination was unremarkable; however, the patient later developed decreased visual acuity bilaterally, with greater impairment on the left side, and lower limb motor strength was also reduced.

**Figure 1:**
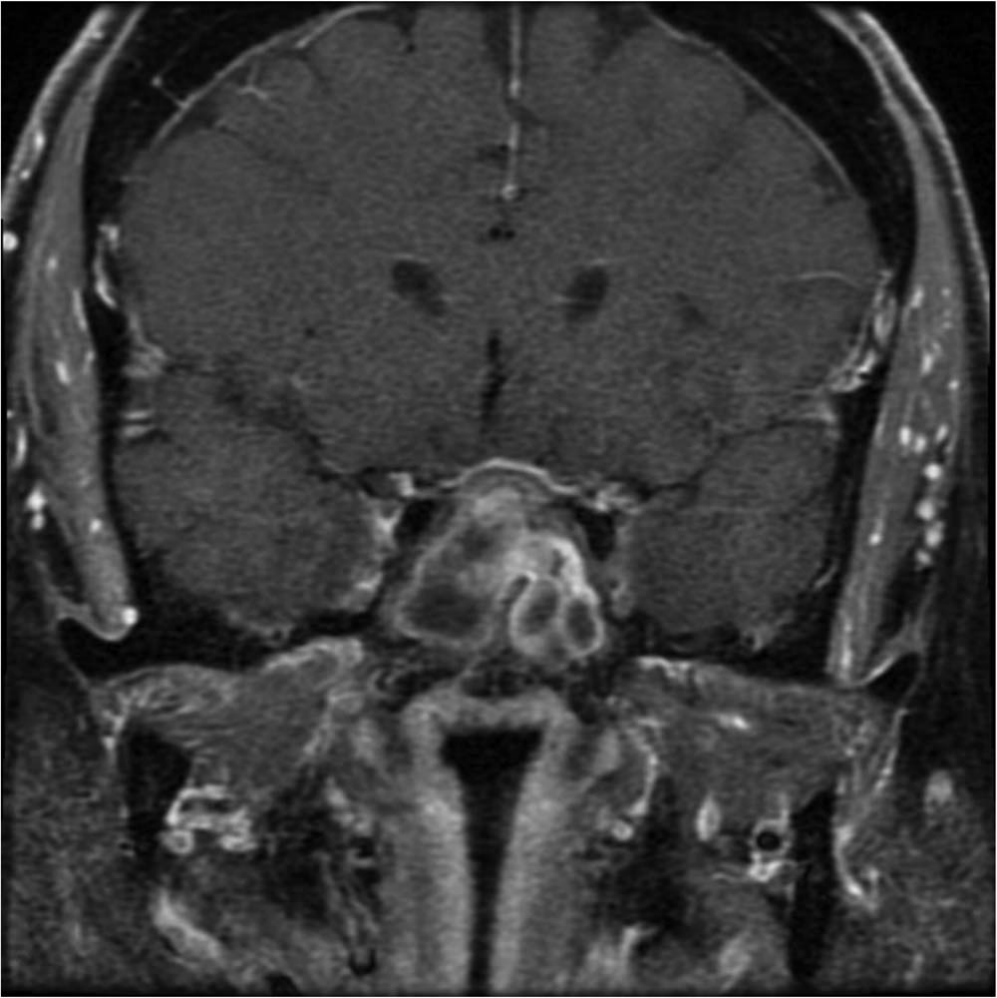
A coronal brain MRI showing a heterogeneous, enhancing lesion located within the sphenoid sinus, extending superiorly to the sellar and parasellar regions. The mass shows irregular borders and appears to cause a mild compression of the optic chiasm.

**Figure 2:**
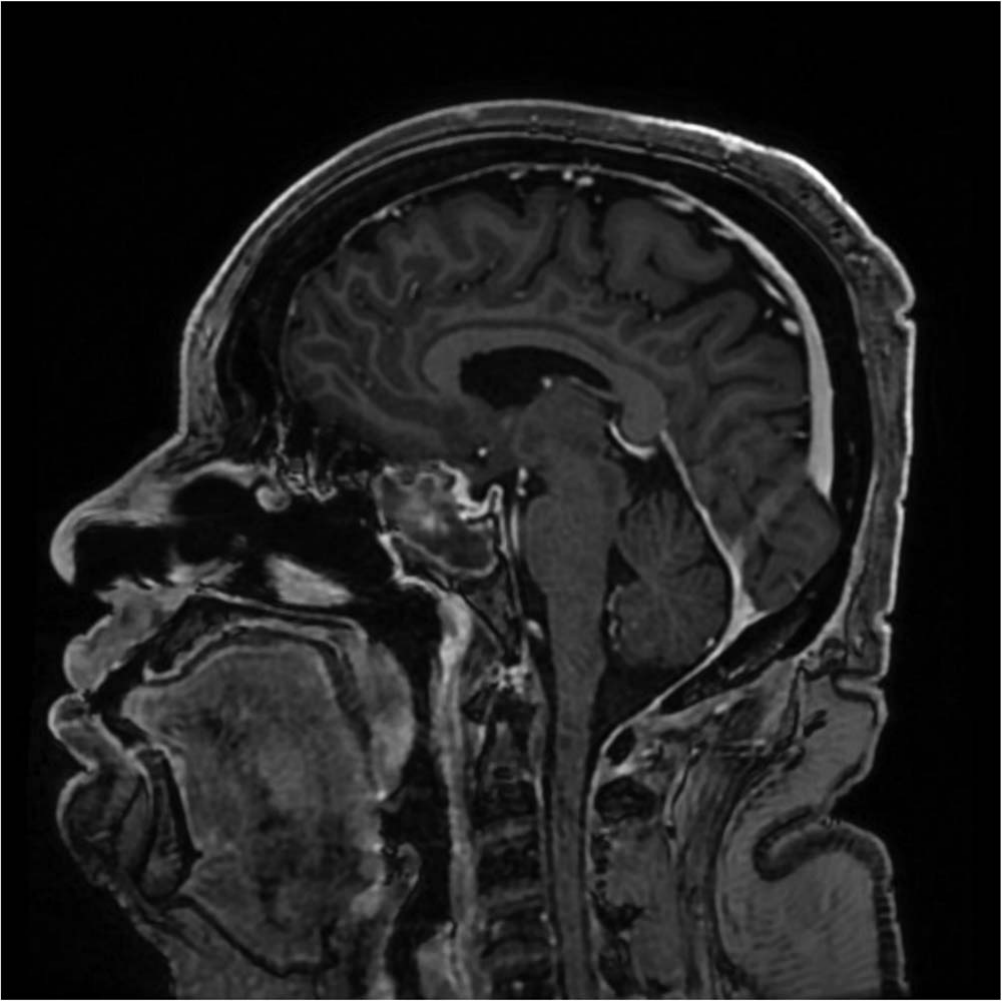
A sagittal brain MRI reveals the same mass with superior extension to the sellar and suprasellar regions. The lesion is exerting a compression to the optic chiasm. The mass effect extends posteriorly toward the clivus, with irregular margins indicating invasive potential. Adjacent structures like the pituitary stalk appear to be displaced.

The initial evaluation included a brain magnetic resonance imaging (MRI), showing a small pituitary mass measuring 1.3 × 2.1 × 1.9 cm with suprasellar extension, neighboring the optic chiasm (Figs 1 and 2). Laboratory results revealed a high prolactin level of 110 ng/ml and normal thyroid function tests. A pituitary macroadenoma, specifically a prolactinoma, was presumed, and cabergoline was initiated.

Despite treatment with cabergoline, follow-up prolactin level remained elevated, and imaging showed a growing tumor size. Clinically, the patient's symptoms persisted, with worsening headaches. Afterward, her prolactin level had dramatically risen to 2000 ng/ml. She was admitted with daily severe headaches, abdominal pain, vomiting, and oral intake intolerance. A brain MRI demonstrated an enlarged sellar and suprasellar mass measuring 3.2 × 1.7 × 3.0 cm, compressing the optic chiasm. Additionally, her prolactin level diminished to 2.7 ng/ml, raising concern of a growing pituitary macroadenoma.

**Figure 3:**
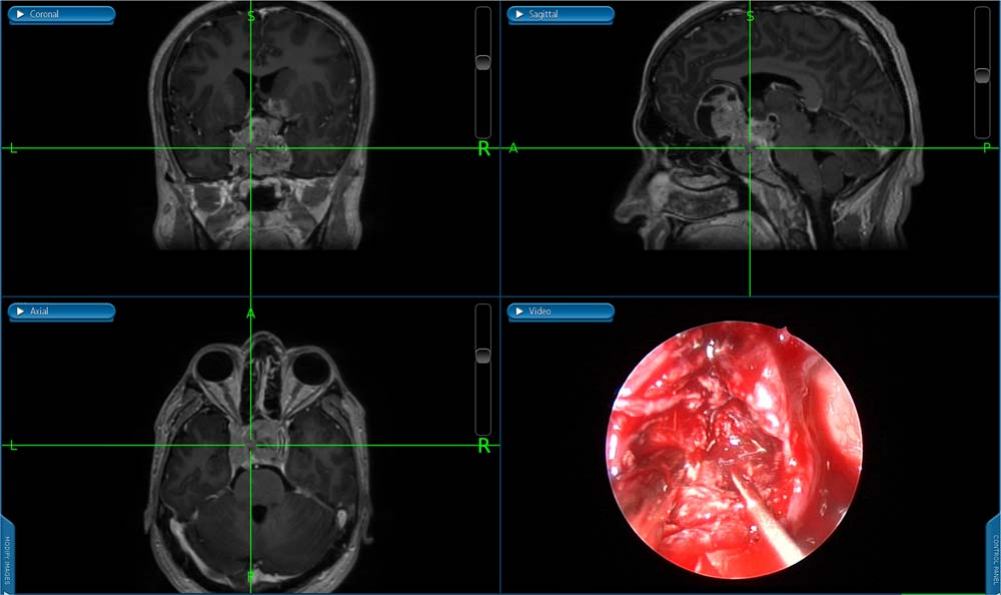
An image-guided intraoperative view of a sellar tumor.

**Figure 4:**
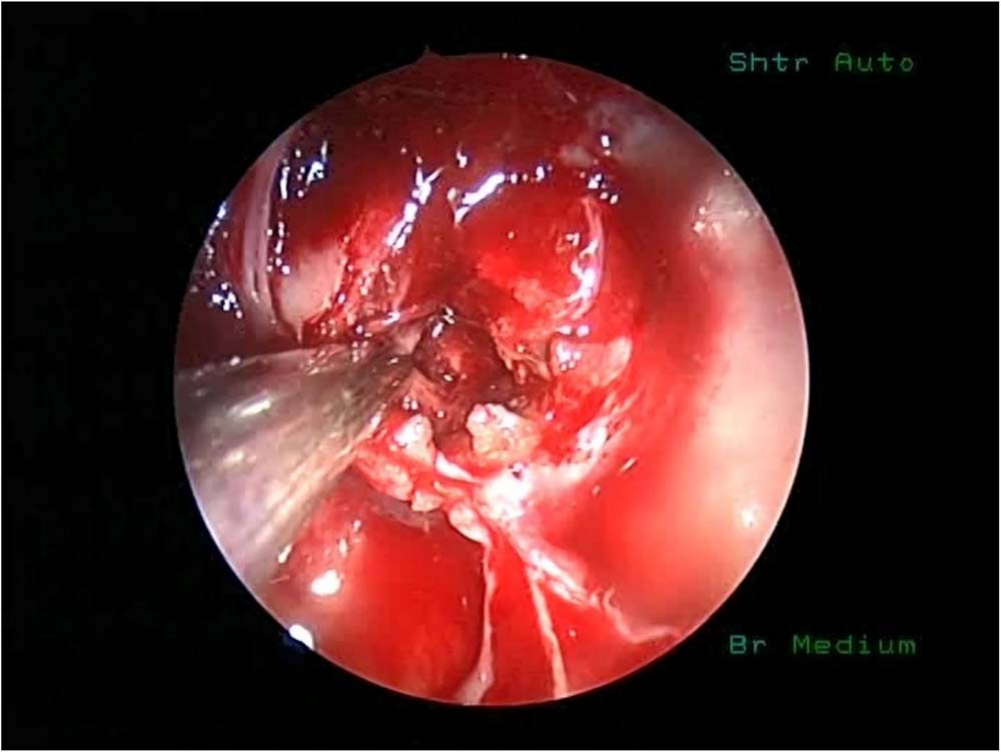
An intraoperative picture of vascular sellar lymphoma.

She underwent endoscopic trans-sphenoidal debulking of the suprasellar lesion (Figs 3 and 4). Postoperatively, the patient developed complications, including diabetes insipidus and panhypopituitarism, requiring levothyroxine and corticosteroid replacement therapy. Histopathology revealed a diagnosis of high-grade primary central nervous system (CNS) B-cell lymphoma. Microscopic analysis showed sheets of atypical lymphoid cells with high mitotic activity. Consequently, the patient was initiated on rituximab and high-dose methotrexate (HD-MTX). Nevertheless, methotrexate was terminated due to the onset of acute kidney injury. Given the patient's intolerance to further HD-MTX, nivolumab was commenced based on PD-L1 expression. While there was partial symptom relief, the tumor response was inadequate. Therefore, the patient underwent whole-brain radiotherapy with a boost to the pituitary region, receiving a total dose of 45 Gy over 25 fractions. This treatment was effective, though she experienced alopecia, mild nausea, blurred vision, headaches, and severe vulvar itching.

During the follow-up appointment, the patient exhibited positive progress, and her nausea was managed with ondansetron. Her MRI demonstrated stable disease with a slight tumor size reduction. She maintained hormone replacement therapy and was scheduled for routine MRI follow-ups. The treatment of endocrine imbalances led to an improvement in her quality of life, coupled by ongoing multidisciplinary support.

## Discussion

A review of the literature demonstrated the difference in characteristics of patients with SSL (Table 1). The age and gender distribution of SSL exhibits variability among the studied cases. The participants comprised younger adults aged 37–45 years, middle-aged adults aged 45–61 years, and older adults aged 61–86 years, representing the majority of reported cases [1–15]. The gender decomposition demonstrated 12 females and 5 males [1–15].

**Table 1:** Summary of reported sellar–suprasellar lymphoma cases

**Study**	**Age, gender**	**Clinical features**	**Endocrine imbalances**	**Imaging finding**	**Histopathology**	**Management**	**Follow-up**
[6]	82, M	Headache, hemianopia, vision loss, and papilledema	Panhypopituitarism	Heterogeneously isointense bilobed tumor enlarging the pituitary fossa with severe compression to the optic chiasm and hypothalamus	DLBCL	Palliative field RT	N/R
[1]	47, F	Worsening headache, blurry vision, right CN III and V palsies, and bitemporal hemianopia	Hyperprolactinemia	Sellar mass with partial involvement of the right internal carotid artery within the cavernous sinus	DLBCL	Surgery, CHT (DeAngelis protocol, R-CHOP, R-ICE), prophylactic intrathecal MTX, autologous stem cell transplantation	Clinical remission following 8 months of stem cell transplantation
[3]	61, M	Headache, unilateral ptosis, unclear vision, anorexia and weakness	Hyperprolactinemia Partial deficiency of anterior pituitary hormones	Sellar mass reaching to the cavernous sinus and right sphenoid	DLBCL	CHT (rituximab, MTX, lenalidomide), intrathecal dexamethasone, and cytarabine	Clinical improvement, mass size reduction following two courses of chemotherapy
[3]	65, F	Headache, N/V, increased urination and thirst, anorexia, and weakness	Panhypopituitarism	Sellar mass which was not differentiated from the optic chiasm	DLBCL	CHT	Died 8 months following diagnosis
[12]	74, M	Bitemporal visual field deficit, worsening weakness, seizure episodes, dizziness with falls, altered mental status, dementia, impotence, constipation	Deficiency of anterior pituitary hormones	Sellar and suprasellar tumor with erosion of the left sellar floor	Large B-cell lymphoma	Surgery, a course of 4600 cGy conventional RT	Died shortly after RT
[12]	65, M	Diplopia, right retrobulbar pain, low energy, and libido	Deficiency of anterior pituitary hormones	Sellar and suprasellar lesion extending into the right cavernous sinus with associated erosion of the sphenoid sinus.	High-grade, large B- cell lymphoma	Surgery, stereotactic radiosurgery and Codox-M of 4 cycles	Died 2 years later due to pulmonary failure
[15]	86, F	Fever spikes, chills, night sweats, and weight loss	Partial deficiency of anterior pituitary hormones	Sellar mass within a slightly enlarged pituitary fossa	B-cell lymphoma	Surgery and CHT	Died 3 months following diagnosis
[13]	41, F	Polyuria, polydipsia, amenorrhea syndrome and galactorrhea	Partial deficiency of anterior pituitary hormones Hyperprolactinemia	Intrasellar and suprasellar mass with thickening of the pituitary stalk and obstructive hydrocephalus	High-grade, large B-cell lymphoma	Surgery, RT (25 fractions of 200 cGy for a total dose of 5000 cGy) and intrathecal MTX	Symptom free for 7 months following management
[2]	69, F	Right frontotemporal headache which developed later to right ptosis, diplopia, and III CN palsy	Hyperprolactinemia High IGF-1 Central hypothyroidism	Large sellar and suprasellar mass with sphenoid bone remodeling with optic chiasm compression and right cavernous sinus invasion	DLBCL	4 cycles of MATRix regimen (methotrexate, cytarabine, thiotepa, and rituximab)	Died of sepsis 3 months later
[9]	45, F	Headache, dizziness, unclear vision, bitemporal hemianopsia, ptosis, numbness of the face	Hyperprolactinemia Partial deficiency of anterior pituitary hormones	Heterogeneous sellar–suprasellar lesion with impingement on the optic nerves and chiasm, and involvement of the cavernous sinuses with thickening of the pituitary stalk	B-cell lymphoma	Surgery, CHT (MTX, vincristine, procarbazine and dexamethasone), R-ICE regimen and autologous transplant	Preserved neurological function 3 months postoperatively, with no signs of disease recurrence.
[4]	61, F	Worsening headaches and acromegaly	High GHRH and IGF-1 Hyperprolactinemia Partial deficiency of anterior pituitary hormones	Isointense, homogenously enhancing sellar tumor without evidence of pituitary stalk displacement	DLBCL, somatotroph hyperplasia	3 cycles of R-CHOP with 3600 cGy of stereotactic RT and 180 cGy for 20 fractions within 27 days	No evidence of mass after 2 months of RT and normalization of prolactin and IGF-1 within 5 months
[10]	37, M	Headaches and progression of visual acuity, bilateral blurred vision, and bitemporal hemianopia	No imbalances	Large intrasellar tumor with a widening of the sella turcica, extending backward into the anteropontal cisterns, which were narrowed, upward enclosing the hypothalamic infundibulum and optic chiasm, cavernous sinus, and sphenoidal involvement	Large B-cell lymphoma	Surgery, 6 cycles of CHOP (cyclophosphamide, vincristine, adriamycin, prednisone), whole-brain RT (dose of 40 Gy and boosted to the tumor 50 Gy)	Stable residual tumor and after 52 months of follow-up, the patient had a mild visual field defect in the right eye.
[11]	59, F	Headache and diplopia	Hyperprolactinemia Partial deficiency of anterior pituitary hormones	Sellar mass extending to the suprasellar cistern and involving the pituitary stalk and cavernous sinuses	High-grade B-cell non-Hodgkin’s lymphoma	No management	Died before starting therapy
[14]	71, F	CN VII palsy, bilateral temporal hemianopsia, syncopal spells, acromegaly	High GH and IGF-1	Well-defined mass extending into the suprasellar cistern, compressing the optic chiasm, and penetrating right cavernous sinus	Large B-cell lymphoma, adenoma, and lymphocytic hypophysitis	Surgery and intensity-modulated RT at dose of 5040 cGy	Complete remission after 1 year of therapy
[5]	73, F	Headache, double vision, retro-orbital pain	Diabetes insipidus	Sellar mass extending to the suprasellar cistern, cavernous sinus, and orbital apex with involvement of the optic chiasm and optic nerve	DLBCL	Surgery, R-CHOP, and high-dose MTX	Improvement after 15 months of RT
[7]	67, F	Headache and left visual field defect	No imbalances	Pituitary mass extending downward to the sphenoid sinus	Diffuse, large, high-grade B-cell pituitary lymphoma	Surgery, stereotactic RT and CHT (4 cycles)	No recurrence after 15 months of follow-ups
[8]	60, F	Weakness, headache, and ptosis	Partial deficiency of anterior pituitary hormones	Pituitary sellar mass extending into the suprasellar region, compressing the optic chiasm, and infiltrating the left cavernous sinus	DLBCL	Surgery, CHT with intraspinal MTX infusion, and R-MPV regimen	Died from hospital-acquired infection
Our case	57, F	Headache, left eye lacrimation, blurry vision, temporal hemianopia, nausea, vomiting, and weight loss	Hyperprolactinemia	Large sellar and suprasellar mass, compressing the optic chiasm, and extending posteriorly toward the clivus with displacement of pituitary stalk	DLBCL	Surgery, rituximab and high-dose MTX which was replaced with nivolumab and whole-brain RT (total dose of 45 Gy over 25 fractions)	Clinically improved with a slight reduction in the mass size

N/R, not reported; DLBCL, diffuse large B-cell lymphoma; RT, radiotherapy; CN, cranial nerve; CHT, chemotherapy; R-CHOP, rituximab-cyclophosphamide, vincristine, adriamycin, prednisone; R-ICE, rituximab, ifosfamide, carboplatin, etoposide; DeAngelis CHT protocol, methotrexate, vincristine, procarbazine, cytarabine; MTX, methotrexate; R-MPV, rituximab, methotrexate, vincristine, and procarvazine.

Clinical manifestations mainly featured neurological and ophthalmological complaints with headaches and visual abnormalities being the most common [1–11]. Several individuals indicated particular visual field impairments, such as bitemporal hemianopsia, diplopia, and ptosis [1, 2, 3, 5–12]. Involvement of cranial nerves, particularly third and fifth nerve palsies was frequently noted and observed along with retrobulbar pain [1, 2, 12]. Furthermore, some cases had weight loss, nausea, vomiting, polydipsia, polyuria, galactorrhea, and cognitive changes as their main presentation [3, 8, 12, 13].

Brain scans like MRI and CT demonstrated sellar or sellar–suprasellar masses of 1–5 cm extending into adjacent structures such as cavernous sinus, sphenoid sinus, or suprasellar cistern, leading to compression of the optic chiasm or hypothalamus [1, 2, 3, 7, 8, 13–15]. Additionally, certain cases featured isointensity or hypointensity on T1- and T2-weighted MRI sequences, accompanied with heterogeneous contrast, frequently resembling a mix of viable mass tissue or pituitary adenomas [1–3, 8, 10]. Certain patients displayed unique bilobed tumors causing hypothalamic compression, a hypermetabolic fludeoxyglucose positron emission tomography/computed tomography (FDG-PET/CT). lesion invading the sphenoid sinus, or dumbbell-shaped masses eroding the sellar floor attributes more indicative of lymphoma [3, 6, 12]. Thickening of the pituitary stalk and invasion of cranial nerves were less common but signified an aggressive disease [11].

Management techniques differed according to the underlying pathology. The primary method to alleviate mass impact and obtain a histological diagnosis was trans-sphenoidal resection, performed using either endoscopic or microsurgical techniques [1, 5, 7–15]. Chemotherapy, including rituximab-cyclophosphamide, vincristine, adriamycin, prednisone (R-CHOP) and methotrexate-based protocols was the main treatment for pituitary lymphomas while radiation therapy (4600–5040 cGy) was used for residual disease [2–4, 6–12, 15]. Other care strategies were documented, including palliative radiation for patients declining aggressive treatment and high-dose glucocorticoids for suspected autoimmune hypophysitis [1, 3]. Furthermore, advanced therapies such as bone marrow transplantation were utilized in a patient with systemic lymphoma involvement [6].

The follow-up results of reported cases revealed a substantial variability. Certain patients achieved remission following chemotherapy and radiotherapy, although others experienced relapses needing salvage chemotherapy [1, 4, 7, 13]. Fatalities had been documented in several cases months after diagnosis [2, 3, 8, 11, 15]. Additionally, a few people needed hormone replacement therapy to treat their endocrine abnormalities [4, 12, 13]. The visual prognosis varied with certain patients attaining complete recovery, while others suffered persistent deficits [2, 10].

## Conclusion

Our case underscores the importance of considering SSL, particularly in patients with unusual sellar masses. Persisting symptoms despite management for pituitary adenoma, accelerated tumor growth, and hormonal abnormalities necessitate detailed imaging and early biopsy. Effective treatment needs a multidisciplinary approach, integrating radiotherapy, and endocrine support when demanded. Early recognition of this rare entity is essential for optimal outcomes.
